# An overview of the antimicrobial resistance mechanisms of bacteria

**DOI:** 10.3934/microbiol.2018.3.482

**Published:** 2018-06-26

**Authors:** Wanda C Reygaert

**Affiliations:** Department of Biomedical Sciences, Oakland University William Beaumont School of Medicine, Rochester, MI, USA

**Keywords:** antimicrobial resistance, β-lactamase, MRSA, ESBL, CRE

## Abstract

Resistance to antimicrobial agents has become a major source of morbidity and mortality worldwide. When antibiotics were first introduced in the 1900's, it was thought that we had won the war against microorganisms. It was soon discovered however, that the microorganisms were capable of developing resistance to any of the drugs that were used. Apparently most pathogenic microorganisms have the capability of developing resistance to at least some antimicrobial agents. The main mechanisms of resistance are: limiting uptake of a drug, modification of a drug target, inactivation of a drug, and active efflux of a drug. These mechanisms may be native to the microorganisms, or acquired from other microorganisms. Understanding more about these mechanisms should hopefully lead to better treatment options for infective diseases, and development of antimicrobial drugs that can withstand the microorganisms attempts to become resistant.

## 1. Introduction

With the discovery of antibiotics, the healthcare community thought that the battle with infectious diseases was won. However, now that so many bacteria have become resistant to multiple antimicrobial agents, the war has seemingly escalated in favor of the bacteria. Infectious diseases are currently a significant cause of morbidity and mortality worldwide. An assessment of these diseases by the World Health Organization (WHO) found that lower respiratory infection, diarrheal diseases, HIV/AIDS, and malaria are in the top ten contributors to morbidity and mortality [1]. The advent of antimicrobial resistance has added significantly to the impact of infectious diseases, in number of infections, as well as added healthcare costs. Even though we have a very large number of antimicrobial agents from which to choose for potential infection therapy, there is documented antimicrobial resistance to all of these, and this resistance occurs shortly after a new drug is okayed for use. These concerns prompted the WHO to launch a Global Action Plan on antimicrobial resistance in 2015 [2].

Antimicrobial agents can be divided into groups based on the mechanism of antimicrobial activity. The main groups are: agents that inhibit cell wall synthesis, depolarize the cell membrane, inhibit protein synthesis, inhibit nuclei acid synthesis, and inhibit metabolic pathways in bacteria. Table 1 gives examples of drugs from each of these groups. It would seem that with such a wide range of mechanisms we would have better control over the organisms. Unfortunately, improper stewardship of antimicrobial agents has helped lead to the tremendous resistance issue that we now face. Factors that have contributed to the growing resistance problem include: increased consumption of antimicrobial drugs, both by humans and animals; and improper prescribing of antimicrobial therapy. Overuse of many common antimicrobials agents by physicians may occur because the choice of drug is based on a combination of low cost and low toxicity [3]. There may also be improper prescribing of antimicrobials drugs, such as the initial prescription of a broad-spectrum drug that is unnecessary, or ultimately found to be ineffective for the organism(s) causing the infection [4]. The danger is that excessive use of antibiotics in humans leads to emergence of resistant organisms [5],[6]. In addition, prior use of antimicrobial drugs puts a patient at risk for infection with a drug resistant organism, and those patients with the highest exposure to antimicrobials are most often those who are infected with resistant bacteria [3],[7].

For many years antibiotics have been used for treating or preventing disease in raising food animals. The animal feed often contains antibiotics in amounts that range from below therapeutic levels to full therapeutic levels, and the antibiotics used come from most of the antimicrobial classes used in humans. There is evidence to support the idea that feeding antibiotics to animals may result in development of antimicrobial resistant organisms, and that those resistant organisms may be transferred to the humans who consume those animals [8],[9]. The antimicrobial resistance patterns seen in the animals reflects the types and amounts of antibiotics given to the animals. The transmission of antimicrobial resistance from the animals to humans may occur in various ways, with the direct oral route being the most common (includes eating meat plus ingestion of feces in contaminated food or water). Another common route is from direct contact with the animals by humans [9].

Continued increases in antimicrobial resistance have led to fewer treatment options for patients, and an associated increase in morbidity and mortality. The result is that now we are facing more severe infections needing more extensive treatment, and longer courses of illness often requiring extended hospitalization. This has dramatically increased the healthcare costs associated with these infections. The CDC has reported that a conservative estimate is that over 2 million people in the U.S become ill each year with antimicrobial resistant infections, resulting in more than 23,000 deaths [10]. The costs attributed to these resistant infections ranges from nearly $7,000 to more than $29,000 per patient [11]. Studies on the healthcare costs for methicillin-resistant *Staphylococcus aureus* (MRSA) infections alone show that in the U.S. the costs are over $18,000 per case, in Germany the costs are nearly €9,000 per case, and in Switzerland there is an average added cost of over 100,000 Swiss francs per case [12]–[14]. Various methods of antimicrobial stewardship have been suggested to stem the increases in resistance. One method involves the use of diversity in antimicrobial use. This refers to various components such as not giving a single drug, but using two or more drugs, either alternatively or concurrently, preferably using drugs with different mechanisms of action [15],[16].

**Table 1.** Antimicrobial groups based on mechanism of action.

Mechanism of Action	Antimicrobial Groups
Inhibit Cell Wall Synthesis	β-Lactams
	Carbapenems
	Cephalosporins
	Monobactams
	Penicillins
	Glycopeptides
Depolarize Cell Membrane	Lipopeptides
Inhibit Protein Synthesis	Bind to 30S Ribosomal Subunit
	Aminoglycosides
	Tetracyclines
	Bind to 50S Ribosomal Subunit
	Chloramphenicol
	Lincosamides
	Macrolides
	Oxazolidinones
	Streptogramins
Inhibit Nucleic Acid Synthesis	Quinolones
	Fluoroquinolones
Inhibit Metabolic Pathways	Sulfonamides
	Trimethoprim

## 2. Persistence versus resistance

**Figure 1.**
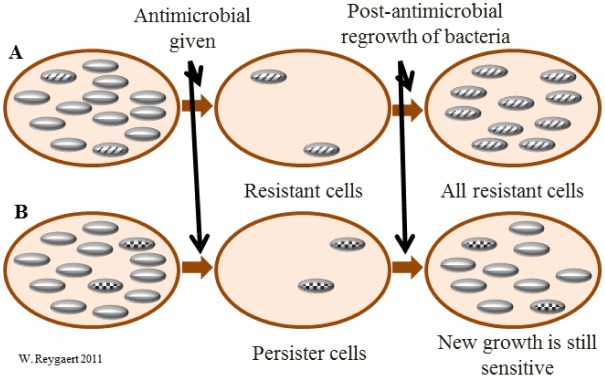
Resistance vs. persistence. When bacterial cells are exposed to an antimicrobial agent there are two possible scenarios. There may be cells present that are resistant to the antimicrobial agent (A). The non-resistant cells are killed, leaving only the resistant cells. When the resistant cells are regrown, all of the cells in the culture will be resistant. The other possibility is that there may be persister cells (dormant, not resistant) present (B). The non-persister cells are killed, leaving only the persister cells. When the persister cells are regrown, those cells not in a dormant state will still be susceptible to the antimicrobial agent.

Before discussing the various aspects of antimicrobial resistance, it would be helpful to distinguish resistance from persistence. If a bacterium is resistant to a certain antimicrobial agent, then all of the daughter cells would also be resistant (unless additional mutations occurred in the meantime). Persistence, however, describes bacterial cells that are not susceptible to the drug, but do not possess resistance genes. The persistence is undoubtedly due to the fact that some cells in a bacterial population may be in stationary growth phase (dormant); and most antimicrobial agents have no effect on cells that are not actively growing and dividing. These persister cells occur at a rate of around 1% in a culture that is in stationary phase [17],[18]. Figure 1 shows the difference between persistent and resistant bacterial cells.

## 3. Origins of resistance

Bacteria as a group or species are not necessarily uniformly susceptible or resistant to any particular antimicrobial agent. Levels of resistance may vary greatly within related bacterial groups. Susceptibility and resistance are usually measured as a function of minimum inhibitory concentration (MIC), the minimal concentration of drug that will inhibit growth of the bacteria. The susceptibility is actually a range of the average MICs for any given drug across the same bacterial species. If that average MIC for a species is in the resistant part of the range, the species is considered to have intrinsic resistance to that drug. Bacteria may also acquire resistance genes from other related organisms, and the level of resistance will vary depending on the species and the genes acquired [19],[20].

### 3.1. Natural resistance

Natural resistance may be intrinsic (always expressed in the species), or induced (the genes are naturally occurring in the bacteria, but are only expressed to resistance levels after exposure to an antibiotic). Intrinsic resistance may be defined as a trait that is shared universally within a bacterial species, is independent of previous antibiotic exposure, and not related to horizontal gene transfer [20],[21]. The most common bacterial mechanisms involved in intrinsic resistance are reduced permeability of the outer membrane (most specifically the lipopolysaccharide, LPS, in gram negative bacteria) and the natural activity of efflux pumps. Multidrug-efflux pumps are also a common mechanism of induced resistance [21],[22]. Table 2 shows some examples of bacteria with intrinsic antimicrobial resistance.

**Table 2.** Examples of bacteria with intrinsic resistance.

Organism	Intrinsic resistance
*Bacteroides* (anaerobes)	aminoglycosides, many β-lactams, quinolones
All gram positives	aztreonam
Enterococci	aminoglycosides, cephalosporins, lincosamides
*Listeria monocytogenes*	cephalosporins
All gram negatives	glycopeptides, lipopeptides
*Escherichia coli*	macrolides
*Klebsiella* spp.	ampicillin
*Serratia marcescens*	macrolides
*Pseudomonas aeruginosa*	sulfonamides, ampicillin, 1^st^ and 2^nd^ generation cephalosporins, chloramphenicol, tetracycline
*Stenotrophomonas maltophilia*	aminoglycosides, β-lactams, carbapenems, quinolones
*Acinetobacter* spp.	ampicillin, glycopeptides

### 3.2. Acquired resistance

Acquisition of genetic material that confers resistance is possible through all of the main routes by which bacteria acquire any genetic material: transformation, transposition, and conjugation (all termed horizontal gene transfer—HGT); plus, the bacteria may experience mutations to its own chromosomal DNA. The acquisition may be temporary or permanent. Plasmid-mediated transmission of resistance genes is the most common route for acquisition of outside genetic material; bacteriophage-borne transmission is fairly rare. Certain bacteria such as *Acinetobacter* spp. are naturally competent, and therefore capable of acquiring genetic material directly from the outside environment. Internally, insertion sequences and integrins may move genetic material around, and stressors (starvation, UV radiation, chemicals, etc.) on the bacteria are common causes of genetic mutations (substitutions, deletions etc.). Bacteria have an average mutation rate of 1 for every 10^6^ to 10^9^ cell divisions, and most of these mutations will be deleterious to the cell [19],[23]. Mutations that aid in antimicrobial resistance usually only occur in a few types of genes; those encoding drug targets, those encoding drug transporters, those encoding regulators that control drug transporters, and those encoding antibiotic-modifying enzymes [20]. In addition, many mutations that confer antimicrobial resistance do so at a cost to the organism. For example, in the acquisition of resistance to methicillin in *Staphylococcus aureus*, the growth rate of the bacteria is significantly decreased [24].

One huge conundrum of antimicrobial resistance is that the use of these drugs leads to increased resistance. Even the use of low or very low concentrations of antimicrobials (sub-inhibitory) can lead to selection of high-level resistance in successive bacterial generations, may select for bacteria that are hypermutatable strains (increase the mutation rate), may increase the ability to acquire resistance to other antimicrobial agents, and may promote the movement of mobile genetic elements [25].

## 4. Mechanisms of resistance

**Figure 2.**
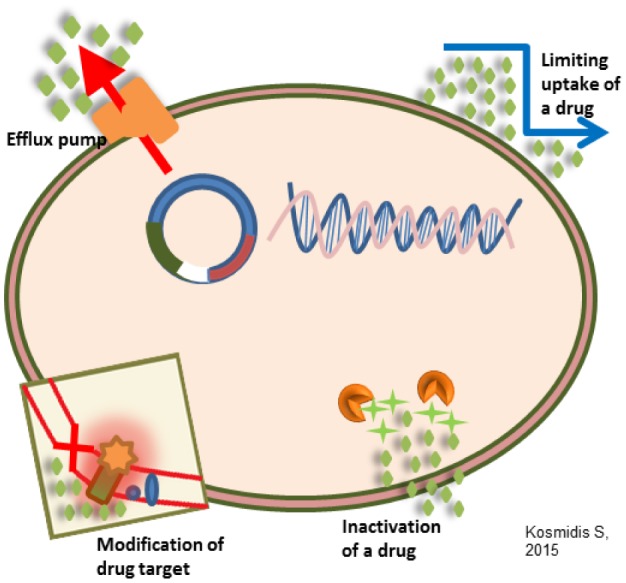
General antimicrobial resistance mechanisms.

Antimicrobial resistance mechanisms fall into four main categories: (1) limiting uptake of a drug; (2) modifying a drug target; (3) inactivating a drug; (4) active drug efflux. Intrinsic resistance may make use of limiting uptake, drug inactivation, and drug efflux; acquired resistance mechanisms used may be drug target modification, drug inactivation, and drug efflux. Because of differences in structure, etc., there is variation in the types of mechanisms used by gram negative bacteria versus gram positive bacteria. Gram negative bacteria make use of all four main mechanisms, whereas gram positive bacteria less commonly use limiting the uptake of a drug (don't have an LPS outer membrane), and don't have the capacity for certain types of drug efflux mechanisms (refer to the drug efflux pumps later in this manuscript) [26],[27]. Figure 2 illustrates the general antimicrobial resistance mechanisms.

### 4.1. Limiting drug uptake

As already mentioned, there is a natural difference in the ability of bacteria to limit the uptake of antimicrobial agents. The structure and functions of the LPS layer in gram negative bacteria provides a barrier to certain types of molecules. This gives those bacteria innate resistance to certain groups of large antimicrobial agents [28]. The mycobacteria have an outer membrane that has a high lipid content, and so hydrophobic drugs such as rifampicin and the fluoroquinolones have an easier access to the cell, but hydrophilic drugs have limited access [29],[30].

Bacteria that lack a cell wall, such as *Mycoplasma* and related species, are therefore intrinsically resistant to all drugs that target the cell wall including β-lactams and glycopeptides [31]. Gram positive bacteria do not possess an outer membrane, and restricting drug access is not as prevalent. In the enterococci, the fact that polar molecules have difficulty penetrating the cell wall gives intrinsic resistance to aminoglycosides. Another gram positive bacteria, *Staphylococcus aureus*, recently has developed resistance to vancomycin. Of the two mechanisms that *S. aureus* uses against vancomycin, a yet unexplained mechanism allows the bacteria to produce a thickened cell wall which makes it difficult for the drug to enter the cell, and provides an intermediate resistance to vancomycin. These strains are designated as VISA strains [30],[32].

In those bacteria with large outer membranes, substances often enter the cell through porin channels. The porin channels in gram negative bacteria generally allow access to hydrophilic molecules [28],[33]. There are two main ways in which porin changes can limit drug uptake: a decrease in the number of porins present, and mutations that change the selectivity of the porin channel [29]. Members of the *Enterobacteriaceae* are known to become resistant due to reducing the number of porins (and sometime stopping production entirely of certain porins). As a group, these bacteria reduce porin number as a mechanism for resistance to carbapenems [34],[35]. Mutations that cause changes within the porin channel have been seen in *E. aerogenes* which then become resistant to imipenem and certain cephalosporins, and in *Neisseria gonorrhoeae* which then become resistant to β-lactams and tetracycline [33],[36].

Another widely seen phenomenon in bacterial colonization is the formation of a biofilm by a bacterial community. These biofilms may contain a predominant organism (such as by *Pseudomonas aeruginosa* in the lung), or may consist of a wide variety of organisms, as seen in the biofilm community of normal flora in the gut. For pathogenic organisms, formation of a biofilm protects the bacteria from attack by the host immune system, plus provides protection from antimicrobial agents. The thick, sticky consistency of the biofilm matrix which contains polysaccharides, and proteins and DNA from the resident bacteria, makes it difficult for antimicrobial agents to reach the bacteria. Thus, to be effective, much higher concentrations of the drugs are necessary. In addition the bacterial cells in the biofilm tend to be sessile (slow metabolism rate, slow cell division), so antimicrobials that target growing, dividing bacterial cells have little effect. An important observation about biofilms is that it is likely that horizontal transfer of genes is facilitated by the proximity of the bacterial cells. That means that sharing of antimicrobial resistance genes is potentially easier for these bacterial communities [37]–[39].

### 4.2. Modification of drug targets

There are multiple components in the bacterial cell that may be targets of antimicrobial agents; and there are just as many targets that may be modified by the bacteria to enable resistance to those drugs. One mechanism of resistance to the β-lactam drugs used almost exclusively by gram positive bacteria is via alterations in the structure and/or number of PBPs (penicillin-binding proteins). PBPs are transpeptidases involved in the construction of peptidoglycan in the cell wall. A change in the number (increase in PBPs that have a decrease in drug binding ability, or decrease in PBPs with normal drug binding) of PBPs impacts the amount of drug that can bind to that target. A change in structure (e.g. PBP2a in *S. aureus* by acquisition of the *mecA* gene) may decrease the ability of the drug to bind, or totally inhibit drug binding [24],[40].

The glycopeptides (e.g. vancomycin) also work by inhibiting cell wall synthesis, and lipopeptides (e.g. daptomycin) work by depolarizing the cell membrane. Gram negative bacteria (thick LPS layer) have intrinsic resistance to these drugs [41]. Resistance to vancomycin has become a major issue in the enterococci (VRE—vancomycin-resistant enterococci) and in *Staphylococcus aureus* (MRSA). Resistance is mediated through acquisition of *van* genes which results in changes in the structure of peptidoglycan precursors that cause a decrease in the binding ability of vancomycin [21],[40]. Daptomycin requires the presence of calcium for binding. Mutations in genes (e.g. *mprF*) change the charge of the cell membrane surface to positive, inhibiting the binding of calcium, and therefore, daptomycin [42]–[44].

Resistance to drugs that target the ribosomal subunits may occur via ribosomal mutation (aminoglycosides, oxazolidinones), ribosomal subunit methylation (aminoglycosides, macrolides—gram positive bacteria, oxazolidinones, streptogramins) most commonly involving *erm* genes, or ribosomal protection (tetracyclines). These mechanisms interfere with the ability of the drug to bind to the ribosome. The level of drug interference varies greatly among these mechanisms [45]–[47].

For drugs that target nucleic acid synthesis (fluoroquinolones), resistance is via modifications in DNA gyrase (gram negative bacteria—e.g. *gyrA*) or topoisomerase IV (gram positive bacteria—e.g. *grlA*). These mutations cause changes in the structure of gyrase and topoisomerase which decrease or eliminate the ability of the drug to bind to these components [48],[49].

For the drugs that inhibit metabolic pathways, resistance is via mutations in enzymes (DHPS—dihydropteroate synthase, DHFR—dihydrofolate reductase) involved in the folate biosynthesis pathway and/or overproduction of resistant DHPS and DHFR enzymes (sulfonamides—DHPS, trimethoprim—DHFR). The sulfonamides and trimethoprim bind to their respective enzymes due to their being structural analogs of the natural substrates (sulfonamides—*p*-amino-benzoic acid, trimethoprim—dihydrofolate). The action of these drugs is through competitive inhibition by binding in the active site of the enzymes. Mutations in these enzymes are most often located in or near the active site, and resulting structural changes in the enzyme interfere with drug binding while still allowing the natural substrate to bind [50],[51].

### 4.3. Drug inactivation

There are two main ways in which bacteria inactivate drugs; by actual degradation of the drug, or by transfer of a chemical group to the drug. The β-lactamases are a very large group of drug hydrolyzing enzymes. Another drug that can be inactivated by hydrolyzation is tetracycline, via the *tetX* gene [45],[52].

Drug inactivation by transfer of a chemical group to the drug most commonly uses transfer of acetyl, phosphoryl, and adenyl groups. There are a large number of transferases that have been identified. Acetylation is the most diversely used mechanism, and is known to be used against the aminoglycosides, chloramphenicol, the streptogramins, and the fluoroquinolones. Phosphorylation and adenylation are known to be used primarily against the aminoglycosides [52]–[55].

### 4.4. β-lactamases

The most widely used group of antimicrobial agents are the β-lactam drugs. The members of this drug group all share a specific core structure which consists of a four-sided β-lactam ring. Resistance to the β-lactam drugs occurs through three general mechanisms: (1) preventing the interaction between the target PBP and the drug, usually by modifying the ability of the drug to bind to the PBP (this is mediated by alterations to existing PBPs or acquisition of other PBPs; (2) the presence of efflux pumps that can extrude β-lactam drugs; (3) hydrolysis of the drug by β-lactamase enzymes [56],[57].

The β-lactamases (originally called penicillinases and cephalosporinases) inactivate β-lactam drugs by hydrolyzing a specific site in the β-lactam ring structure, causing the ring to open. The open-ring drugs are not able to bind to their target PBP proteins. The known β-lactamases are wide-spread, and the group contains enzymes that are able to inactivate any of the current β-lactam drugs. The production of β-lactamases is the most common resistance mechanism used by gram negative bacteria against β-lactam drugs, and the most important resistance mechanism against penicillin and cephalosporin drugs [45],[58].

The β-lactamase enzymes are classified based on their molecular structure and/or functional characteristics. Structurally they are placed into four main categories (A, B, C, or D). There are three functional groupings based on the substrate specificity: the cephalosporinases, the serine β-lactamases, and the metallo (zinc-dependent) β-lactamases. These enzymes may also be commonly known by their enzyme family; for example: the TEM (named after the first patient) family, the SHV (sulphydryl variable) family, and the CTX (preferentially hydrolyze cefotaxime) family. Gram negative bacteria may produce β-lactamases from all four structural groups. The β-lactamases found in gram positive bacteria are mainly from group A, with some from group B [59]–[62].

These enzymes may be innately found on the bacterial chromosome or may be acquired via a plasmid. Many members of the *Enterobacteriaceae* family of gram negative bacteria possess chromosomal β-lactamase genes. Other gram negative bacteria that possess these include *Aeromonas* spp., *Acinetobacter* spp., and *Pseudomonas* spp. Plasmid-carried β-lactamase genes are most commonly found in the *Enterobacteriaceae*, but may also be found in some species of gram positive bacteria such as *Staphylococcus aureus*, *Enterococcus faecalis*, and *Enterococcus faecium* [26],[59].

The first β-lactamase to be characterized was from *E. coli* and is chromosomally encoded by the *ampC* gene (so named for ampicillin resistance). This gene is constitutively expressed at a low level, but mutations may result in overexpression of the gene. The AmpC β-lactamases are most effective against the penicillins and some first generation cephalosporins. There are also many plasmid-borne β-lactamases which carry a variety of *bla* genes (β-lactamase genes). If these β-lactamases confer resistance to later generation cephalosporins, they were designated as ESBLs, and include members of the TEM, SHV, CTX-M, and OXA enzyme families. The largest group is the CTX-Ms, which are most commonly found in *E. coli*, especially UTI isolates. The ESBL producers may also be resistant to multiple drug classes, but are generally sensitive to β-lactamase inhibitors. The β-lactamase inhibitors are structurally similar to β-lactamases, have weak antimicrobial ability alone, but work synergistically in combination with a β-lactam drug. Commonly used β-lactamase inhibitor/drug pairings include amoxicillin/clavulanic acid, ampicillin/sulbactam, and piperacillin/tazobactam [56],[59],[60],[63]–[66].

Recently there has been an emergence of β-lactamases that are active against the carbapenems (carbapenemases), and are found primarily in the *Enterobacteriaceae*. There are two types of carbapenemases; the *Klebsiella pneumoniae* carbapenemases (KPCs), and those designated as Carbapenem-Resistant Enterobacteriaceae (CRE) enzymes. The KPCs belong to the serine Class A (functional group 2f) β-lactamases, are resistant to all β-lactam drugs, but may still be affected by β-lactamase inhibitors. In bacteria that are CRE strains the carbapenemases are all metallo-β-lactamases (MBLs) in Class B, functional group 3a, and are capable of hydrolyzing all β-lactam drugs, but are not inactivated by β-lactamase inhibitors. The most widely distributed CREs are the IMP-1 (for imipenem resistance) and VIM-1 (Verona integron encoded MBL) types. A new MBL has recently been identified, mainly in strains of *E. coli*. It has been designated as NDM-1 (New Delhi MBL). Infections caused by CRE strains have been associated with in-hospital mortality of up to 71% [56]–[58],[67],[68].

There is a lot of emphasis on the development of more effective β-lactamase inhibitor drug combinations, especially in an effort to combat the CRE strains. One newer β-lactamase/drug combination is ceftolozane/tazobactam, which is mainly used against *P. aeruginosa*, and shows promise against gram negative ESBL producing strains. There are also newer β-lactamase inhibitors which do not have a structure similar to the β-lactam drugs. The first one of these to be approved for use is avibactam, and it has been approved for use with ceftazidime against gram negative bacteria. In addition, avibactam is being tested for use with aztreonam against CREs. Another β-lactamase inhibitor which in non β-lactam structured is vaborbactam. It was approved for use with meropenem in 2017 against gram negative bacteria causing complicated urinary tract infections (cUTIs). Unfortunately, so far none of the newer combination drugs is designed to combat the CREs directly. The metallo-β-lactamases are proving difficult to defeat as these enzymes comprise 3 groups that vary greatly in structure and mechanisms [69]–[71].

### 4.5. Drug efflux

Bacteria possess chromosomally encoded genes for efflux pumps. Some are expressed constitutively, and others are induced or overexpressed (high-level resistance is usually via a mutation that modifies the transport channel) under certain environmental stimuli or when a suitable substrate is present. The efflux pumps function primarily to rid the bacterial cell of toxic substances, and many of these pumps will transport a large variety of compounds (multi-drug [MDR] efflux pumps). The resistance capability of many of these pumps is influenced by what carbon source is available [28],[72].

**Figure 3.**
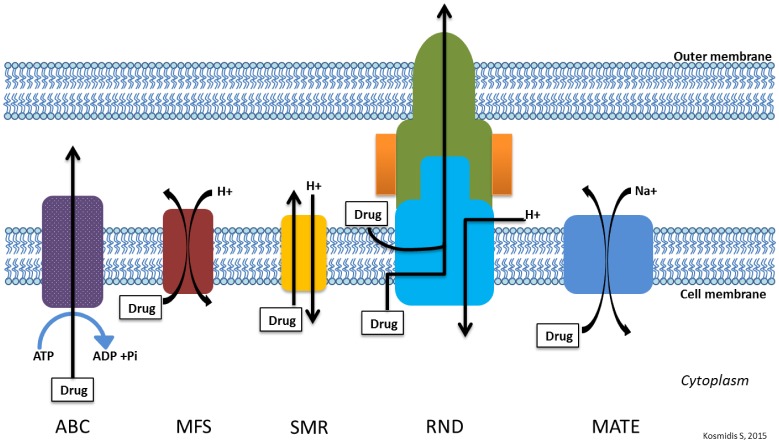
General structure of main efflux pump families.

Most bacteria possess many different types of efflux pumps. There are five main families of efflux pumps in bacteria classified based on structure and energy source: the ATP-binding cassette (ABC) family, the multidrug and toxic compound extrusion (MATE) family, the small multidrug resistance (SMR) family, the major facilitator superfamily (MFS), and the resistance-nodulation-cell division (RND) family. Most of these efflux pump families are single-component pumps which transport substrates across the cytoplasmic membrane. The RND family are multi-component pumps (found almost exclusively in gram negative bacteria) that function in association with a periplasmic membrane fusion protein (MFP) and an outer membrane protein (OMP-porin) to efflux substrate across the entire cell envelope [28],[29],[73],[74]. There are instances where other efflux family members act with other cellular components as multicomponent pumps in gram negative bacteria. One member of the ABC family, MacB, works as a tripartite pump (MacAB-TolC) to extrude macrolide drugs. A member of the MFS, EmrB, works as a tripartite pump (EmrAB-TolC) to extrude nalidixic acid in *E. coli* [75],[76]. Figure 3 shows the basic structure of the various efflux pump families.

Efflux pumps found in gram positive bacteria may confer intrinsic resistance because of being encoded on the chromosome. These pumps include members of the MATE and MFS families and efflux fluoroquinolones. There are also gram positive efflux pumps known to be carried on plasmids. Currently, the characterized pumps in gram positive bacteria are from the MFS family [77]–[80]. Efflux pumps found in gram negative bacteria are widely distributed and may come from all five of the families, with the most clinically significant pumps belonging to the RND family [28],[79].

### 4.6. ABC transporter family

The ABC efflux family contains both uptake and efflux transport systems. The members of this family are unique in that they use energy derived from ATP hydrolysis. These pumps transport amino acids, drugs, ions, polysaccharides, proteins, and sugars. Bacterial ABC transporters usually are made up of six transmembrane segments (TMS) consisting of α-helices, function in the membrane in pairs, either as homodimers or heterodimers, and work in conjunction with cytoplasmic ATPases. These pumps have fairly specific substrates, and there are very few found in clinically significant bacteria. One notable ABC pump is found in *Vibrio cholerae* (VcaM), and is capable of transporting fluoroquinolones and tetracycline [29],[81],[82].

### 4.7. MATE transporter family

The MATE efflux family use a Na^+^ gradient as the energy source, and efflux cationic dyes, and most efflux fluoroquinolone drugs. Some MATE pumps have also been shown to efflux some aminoglycosides. Other substrates for these pumps may have unrelated chemical structures. These pumps are made up of twelve TMS. Very few of these have been characterized in bacteria, and most are found in gram negative organisms. The first to be characterized was the NorM pump from chromosomal DNA in *Vibrio parahaemolyticus*. Other clinically significant bacteria that have NorM pumps include *Neisseria gonorrhoeae* and *Neisseria meningitidis* [73],[83],[84].

### 4.8. SMR transporter family

The SMR efflux family are energized by the proton-motive force (H^+^), are hydrophobic, and efflux mainly lipophilic cations, so may have a very narrow substrate range. The genes for these pumps have been found in chromosomal DNA and on plasmids and transposable elements. These pumps are made up of four TMS and function as asymmetrical homotetramers. Drug efflux has only been seen in a few of these pumps, and these most commonly confer resistance to β-lactams and some aminoglycosides. Examples of SMR pumps are seen in *Staphylococcus epidermidis* (the SMR pump which transports ampicillin, erythromycin and tetracycline) and *Escherichia coli* (the EmeR pump which transports vancomycin, erythromycin, and tetracycline) [28],[29],[85],[86].

### 4.9. MFS transporter family

The MFS efflux family catalyze transport via solute/cation (H^+^ or Na^+^) symport or solute/H^+^ antiport. They are involved in the transport of anions, drugs (e.g. macrolides and tetracycline), metabolites (e.g. bile salts), and sugars. The MFS pumps have the greatest substrate diversity as a group, yet individually tend to be substrate specific. Examples of this substrate specificity include *Acinetobacter baumannii* having separate MFS pumps for erythromycin (SmvA) and chloramphenicol (CraA and CmlA), and *Escherichia coli* having separate MFS pumps for macrolides (MefB), fluoroquinolones (QepA), and trimethoprim (Fsr). There are rare examples of MFS pumps with a slightly broader substrate specificity, such as in the NorA pump in *Staphylococcus aureus* which transports fluoroquinolones and chloramphenicol (these antimicrobials are the most commonly transported by MFS pumps), or the *S. aureus* LmrS pump which transports linezolid, erythromycin, chloramphenicol, and trimethoprim. These pumps are made up of twelve or fourteen TMS, and over 1,000 have been sequenced in bacteria. Most MFS pumps have been found on bacterial chromosomes, and nearly 50% of the efflux pumps in *E. coli* are MFS pumps [28],[29],[45],[87].

### 4.10. RND transporter family

The RND efflux family members catalyze substrate efflux via a substrate/H^+^ antiport mechanism, and are found in numerous gram negative bacteria. They are involved in the efflux of antibiotics (all are multi-drug transporters), detergents, dyes, heavy metals, solvents, and many other substrates. Some of these pumps may be drug or drug class specific (Tet pump—tetracycline; Mef pump—macrolides). Many other RND pumps are capable of transporting a wide range of drugs, such as the MexAB-OprM pump in *Pseudomonas aeruginosa* that confers intrinsic resistance to β-lactams, chloramphenicol, tetracycline, trimethoprim, sulfamethoxazole, and some fluoroquinolones. These pumps are complex multi-component pumps generally made up of twelve TMS and contain two large periplasmic loops between TMS 1 and 2, and TMS 7 and 8. In order to function, these pumps will connect to an OMP and that connection is stabilized by MFPs. Interestingly, these pumps share a high degree of homology among the RND members. The genes for the RND pumps are generally organized as an operon. In many, the gene organization is as follows: the gene for the regulator (which may be transcribed in the opposite direction to the other genes) is adjacent to the MFP gene, which is adjacent to the main pump gene, and then the OMP gene. Probably the most widely studied RND pump is the AcrAB-TolC pump in *Escherichia coli*, which confers resistance to penicillins, chloramphenicol, macrolides, fluoroquinolones, and tetracycline. The AcrB pump protein contains two binding pockets which allow the binding of substrates of varying size and chemical properties [28],[29],[52],[73],[74],[79],[82],[88].

Table 3 shows a summary of the antimicrobial resistance mechanisms that are used against the various drugs.

**Table 3.** Antimicrobial resistance mechanisms.

Drug	Drug Uptake Limitation	Drug Target Modification	Drug Inactivation	Efflux Pumps
β-Lactams	Decreased numbers of porins, no outer cell wall	Gram pos—alterations in PBPs	Gram pos, gram neg—β-lactamases	RND
Carbapenems	Changed selectivity of porin			
Cephalosporins	Changed selectivity of porin			
Monobactams				
Penicillins				
Glycopeptides	Thickened cell wall, no outer cell wall	Modified peptidoglycan
Lipopeptides		Modified net cell surface charge		
Aminoglycosides	Cell wall polarity	Ribosomal mutation, methylation	Aminoglycoside modifying enzymes, acetylation, phosphorylation, adenylation	RND
Tetracyclines	Decreased numbers of porins	Ribosomal protection	Antibiotic modification, oxidation	MFS, RND
Chloramphenicol		Ribosomal methylation	Acetylation of drug	MFS, RND
Lincosamides		Gram pos—ribosomal methylation		ABC, RND
Macrolides		Ribosomal mutation, methylation		ABC, MFS, RND
Oxazolidinones		Ribosomal methylation		RND
Streptogramins				ABC
Fluoroquinolones		Gram neg—DNA gyrase modification	Acetylation of drug	MATE, MFS, RND
		Gram pos—topoisomerase IV
Sulfonamides		DHPS reduced binding, overproduction of resistant DHPS		RND
Trimethoprim		DHFR reduced binding, overproduction of DHFR		RND

ABC—ATP binding cassette family, DHFR—dihydrofolate reductase, DHPS—dihydropteroate synthase, MATE—multidrug and toxic compound extrusion family, MFS—major facilitator superfamily, PBP—penicillin-binding protein, RND—resistance-nodulation-cell division family.

### 4.11. Impact of antimicrobial resistance for individual bacteria

It is vitally important that we have a clear picture of how many of these resistance mechanisms individual bacteria may have in their arsenals. An excellent and important example of this is MRSA. The increase in costs for MRSA infections was mentioned previously [12]–[14]. These increased costs are affected by excess length of hospital stay, increases in number of tests needed, and increased medical and rehabilitation services provided. We also need to think about the impact on morbidity and mortality caused by MRSA, including significant increases in disease complications. The methicillin susceptible *Staphylococcus aureus* (MSSA) and MRSA strains possess the same large number of virulence factors including surface molecules that promote colonization, and secreted molecules that allow invasion of and damage to host cells. These virulence factors assist the bacteria in causing multiple types of infections. Since MRSA is well known for infections of skin and related tissues, it is easier to spread the infection from person-to-person, especially in hospital settings. It has been estimated that the mortality rate for MRSA infections is 2–3 times higher than that for MSSA strains. In addition, MRSA strains are frequently multidrug resistant, which limits the impact of available antimicrobial therapy [24],[61]. Table 4 is a summary of the types of resistance mechanisms that *S. aureus* has in place [61]. There are of course, many pathogens that have similarly diverse arsenals (e.g. *Escherichia coli* and *Klebsiella pneumoniae*) and are becoming resistant to most of the antimicrobial agents available.

**Table 4.** Antimicrobial resistance mechanisms in *Staphylococcus aureus*.

Resistance Mechanism	Antimicrobial Agents
Limiting Drug Uptake	Glycopeptides
Modification of Drug Target	*β*-lactams
	Glycopeptides
	Lipopeptides
	Aminoglycosides
	Tetracyclines
	Macrolides
	Lincosamides
	Oxazolidinones
	Streptogramins
	Fluoroquinolones
	Metabolic Pathway Inhibitors
Inactivation of Drug	*β*-lactams
	Chloramphenicol
Active Drug Efflux	Tetracyclines
	Fluoroquinolones

## 5. Conclusions

The reality is that bacterial are very versatile and adaptive. In order to survive they need to be capable of dealing with toxic substances. Free living bacteria need to be able to survive toxic attacks and waste products from other organisms. It should come as no surprise that the bacteria that infect humans are able to defend themselves against antimicrobial agents. With the alarming increase in antimicrobial resistance, it is imperative that we find ways to combat these pathogens. Unfortunately, there is no easy (or cheap, probably) answer to this dilemma. Perhaps we need to rethink how we design new antimicrobial agents; or maybe start looking to natural substances for clues on what could be used in this fight.

The mechanisms described here are as varied as are the bacteria themselves. These bacterial weapons pretty much cover all of the antimicrobial agents that we have, and there are probably more resistance mechanisms out there that we have not yet characterized. The outlook for fighting microorganisms might seem to be a little bleak. In 2010 the Infectious Diseases Society of America (ISDA) requested that by 2020 there would be FDA approval of 10 novel antibiotics. As of 2016, 8 new drugs had been approved, but only one of these is a novel antibiotic. The median time in the approval pipeline for these drugs was 6.2 years, and the cost per dose of these drugs ranges from nearly $2,000 to nearly $4,200 [89]. So we will need to work hard, and work quickly to find remedies for this pressing problem.
